# Nanoparticle-based delivery systems as emerging therapy in retinoblastoma: recent advances, challenges and prospects

**DOI:** 10.1039/d3na00462g

**Published:** 2023-08-15

**Authors:** Adaeze Linda Onugwu, Onyinyechi Lydia Ugorji, Chinasa A. Ufondu, Stella Amarachi Ihim, Adaeze Chidiebere Echezona, Chinekwu Sherridan Nwagwu, Sabastine Obinna Onugwu, Samuel WisdomofGod Uzondu, Chinazom Precious Agbo, John Dike Ogbonna, Anthony Amaechi Attama

**Affiliations:** a Drug Delivery and Nanomedicines Research Laboratory, Department of Pharmaceutics, University of Nigeria Nsukka Enugu State Nigeria adaeze.onugwu@unn.edu.ng anthony.attama@unn.edu.ng; b Department of Pharmaceutical Technology and Industrial Pharmacy, University of Nigeria Nsukka Enugu State Nigeria; c Molecular Pharmacology and Therapeutics, Department of Pharmacology, University of Minnesota Twin Cities USA; d Department of Science Laboratory Technology (Physiology and Pharmacology Unit), University of Nigeria Nsukka Enugu State Nigeria; e Department of Pharmacognosy, Enugu State University of Science and Technology Enugu State Nigeria; f NanoMalaria Research Unit, Drug Delivery and Nanomedicines Research Laboratory, Department of Pharmaceutics, University of Nigeria Nsukka Enugu State Nigeria; g Institute for Drug-Herbal Medicine-Excipient Research and Development, University of Nigeria Nsukka Enugu State Nigeria

## Abstract

Retinoblastoma is the most common intraocular malignancy in children. The treatment of this rare disease is still challenging in developing countries due to delayed diagnosis. The current therapies comprise mainly surgery, radiotherapy and chemotherapy. The adverse effects of radiation and chemotherapeutic drugs have been reported to contribute to the high mortality rate and affect patients' quality of life. The systemic side effects resulting from the distribution of chemotherapeutic drugs to non-cancerous cells are enormous and have been recognized as one of the reasons why most potent anticancer compounds fail in clinical trials. Nanoparticulate delivery systems have the potential to revolutionize cancer treatment by offering targeted delivery, enhanced penetration and retention effects, increased bioavailability, and an improved toxicity profile. Notwithstanding the plethora of evidence on the beneficial effects of nanoparticles in retinoblastoma, the clinical translation of this carrier is yet to be given the needed attention. This paper reviews the current and emerging treatment options for retinoblastoma, with emphasis on recent investigations on the use of various classes of nanoparticles in diagnosing and treating retinoblastoma. It also presents the use of ligand-conjugated and smart nanoparticles in the active targeting of anticancer and imaging agents to the tumour cells. In addition, this review discusses the prospects and challenges in translating this nanocarrier into clinical use for retinoblastoma therapy. This review may provide new insight for formulation scientists to explore in order to facilitate the development of more effective and safer medicines for children suffering from retinoblastoma.

## Abstract 2

Nanoparticles utilized in targeting retinoblastoma cells demonstrated improved uptake and intracellular internalization, sustained retention, excellent cytotoxicity, enhanced apoptosis and improved antitumor activity.

## Graphical abstract

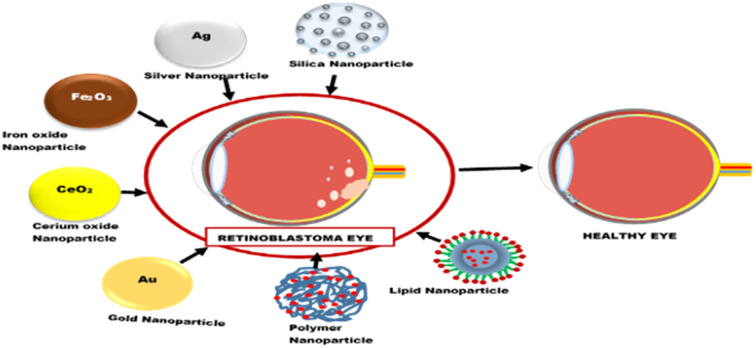

## 1. Introduction

Cancer is a major contributor to the world's mortality rate, as almost 10 million deaths were reported in the year 2020.^1^ The disease and conventional treatment options have enormous adverse effects on the patient's quality of life. Cancers affect different parts of the body, including the breast, lungs, prostate, skin and eyes. Retinoblastoma is a type of intraocular cancer resulting from a mutation in both alleles of the retinoblastoma RB1 tumour suppressor gene (RB1) (Ch13q14.2) in the developing retinal progenitor cells.^2^ The reduction in the expression of the tumour suppressor gene eradicates the control in the cell cycle and leads to the unregulated proliferation of cells. About 40% of retinoblastomas are heritable, while 60% occur sporadically. Unlike the non-heritable cases, heritable retinoblastomas present earlier, are bilateral and multifocal, and the patients have an increased risk of developing secondary malignancies.

Retinoblastoma is the most common primary intraocular malignancy in children, representing 4% of all childhood tumours with an average incidence of 1 in 17 000 births.^3^ More than 9 out of 10 pediatric cases are presented before their fifth birthday.^3^ The most common symptoms of retinoblastoma are leukocoria (white pupil) and strabismus (misaligned eyes). Other symptoms can be seen in advanced retinoblastomas, such as a colour change in the iris and abnormal bulging out of the eyes.^4^

**Fig. 1:**
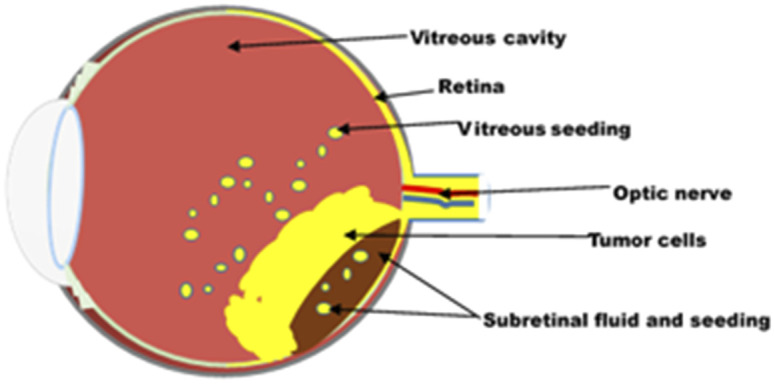
Retinoblastoma eye.

The prognosis of RB is excellent if diagnosed early and treated aggressively with cure rates greater than 90%. However, RB remains a potentially deadly disease in developing countries because of late diagnosis and poor healthcare systems.^5^ With a delayed diagnosis, the tumour expands towards the vitreous humour, subretinal space and brain *via* the optic nerve (Fig. 1). It then metastasizes to other organs of the body, such as bone and liver, with disastrous outcomes including blindness, secondary tumours and death. Therefore, early diagnosis and effective treatment options are crucial for successful treatment.

Retinoblastoma therapy aims to prevent metastasis, decrease the risk of secondary tumours, salvage the eye and preserve vision.

Retinoblastoma is conventionally treated by surgery (enucleation), brachytherapy, external beam radiotherapy, cryotherapy, thermotherapy, photocoagulation, systemic chemotherapy, intra-arterial or intravitreal chemotherapy or combination therapy. Brachytherapy, cryotherapy, thermotherapy and photocoagulation are the focal therapies employed in the early stage of cancer when the tumour is still small and restricted to the retina. However, they affect healthy cells of the eye leading to complications such as retinal traction and detachment, cataracts, retinal fibrosis, vitreoretinopathy, iris atrophy and chorioretinal atrophy.^6–8^ External beam radiotherapy is rarely used because of the risk of a second cancer in patients with heritable retinoblastoma, cataract and toxic effects on ocular tissues.^9^

The advanced stage of retinoblastoma, usually characterized by larger tumours, vitreous and subretinal seeds, extraocular involvement, and metastasis, is managed with chemotherapy and surgery. The treatment is then consolidated with focal therapy. Chemotherapy utilizes cytotoxic agents to destroy cancer cells. Chemotherapeutic agents can be administered systemically (intravenous) or locally (intra-arterial or intravitreal). Commonly used anticancer drugs are carboplatin, cisplatin, etoposide, vincristine, topotecan and melphalan. When administered systemically, these chemotherapeutic agents, due to their non-specific and non-selective nature, cause organ damage and other adverse effects, including transient neutropenia, hyponatremia, hepatotoxicity, neurotoxicity, nephrotoxicity and ototoxicity.^10^ It is also challenging for the chemotherapeutic drug to pass the blood–ocular barriers to reach the target site. The development of the intra-arterial route to deliver cytotoxic drugs directly to the eye has improved the therapeutic outcome of retinoblastoma.^11–13^ Most importantly, the advent of intravitreal chemotherapy involving the injection of drugs directly into the vitreous cavity has yielded excellent outcomes in eliminating vitreous and subretinal tumour seeding. Local chemotherapy through the above routes has tremendously helped salvage the eye in patients with advanced retinoblastoma.^14–17^ However, these procedures are invasive and require skilled personnel. Furthermore, complications such as vitreous haemorrhage, retinal pigment epithelial alterations, pulmonary toxicity, arrhythmias and retinal vasculitis have been reported.^18,19^ They are also expensive and inaccessible to patients in developing countries. Hence, surgery is still the mainstay therapy for advanced retinoblastoma in those countries. Enucleation is non-conservative and results in permanent loss of the eye and facial deformity.

Despite significant improvement in retinoblastoma treatment due to the increasing use of local chemotherapy and focal therapy, a significant safety concern always arises about the adverse side effects and toxicity caused by chemotherapeutic drugs and radiation. The toxic effects caused by these therapies on healthy tissues and organs have been reported to cause high mortality rates in cancer patients.^20,21^ The emerging treatments being investigated include new or repositioned drugs, less invasive routes for local drug delivery such as subconjunctival, subtenon, intracameral and intrathecal routes, photodynamic therapy, immunotherapy, novel delivery systems, active targeting using functionalized carriers or smart systems, and molecularly targeted therapies.^22–27^ Newer radiation-based therapies, such as intensity-modulated radiation therapy and proton beam therapy, aim to target the tumour specifically and reduce off-target effects.

Nanotechnology-based drug delivery systems have been proposed as safe and efficient carriers for chemotherapeutic agents in the treatment of various cancers. Cocarta *et al.* developed a transscleral two-layered hydrogel implant to overcome the off-target toxicity and increase the efficacy of topotecan and vincristine.^28^ The biocompatible implant demonstrated significant cytotoxicity against RB cells. Based on the findings, the hydrogel implants loaded with topotecan and vincristine showed great potential for the local therapy of retinoblastoma. In a recent study, Wang *et al.* designed a biosynthetic protein-based nanocarrier to improve the delivery of etoposide and minimize its adverse effects. There was significant enhancement of the solubility, release, cellular uptake and antitumor activity of the drug. The authors recorded a controlled release of etoposide lasting for six days. Most importantly, there was a significant reduction in the hepatotoxicity and nephrotoxicity of the drug.^29^

An emerging area in cancer therapy is the use of smart nanocarriers. These nanocarriers respond to internal or external stimuli to release their cargo at specific tumour sites.^30^ Salas-Treviño *et al.* investigated hyaluronic acid-modified carbon nanotubes as a novel delivery system for carboplatin. Due to the affinity of hyaluronic acid with CD44 receptors overexpressed by tumour cells, a significant amount of the nanocarriers was taken up by lung cancer cells *via* CD44-mediated endocytosis.^31^ The internalization of the drug by the tumour cells led to enhanced cytotoxic effects.

Nanocarriers have also been reported to enhance the efficiency of imaging modalities. This is crucial as proper evaluation of retinoblastoma patients is paramount to effective treatment. Na *et al.* developed an imaging agent, gadolinium, in glycol chitosan nanoparticles for targeted imaging of liver tumours.^32^ More efficient MR images were obtained with the gadolinium-loaded nanoparticles compared with the commercial control. Furthermore, the mice injected with the nanocarriers recorded no adverse effects, while those administered with the control exhibited liver and kidney damage. Hence, gadolinium-loaded glycol chitosan nanoparticles were safer and more efficient as a T1-weighted magnetic resonance imaging agent.

The use of nanoparticles has been extensively investigated in the diagnosis and treatment of ocular diseases.^33,34^ Their nano size, morphology, and surface characteristics facilitate their entry into ocular tissue, bypassing the ocular barriers and improving their bioavailability and therapeutic efficacy.^33^ They offer the advantages of controlled, sustained and targeted drug delivery with minimum side effects.^35–37^ Several researchers have reported the potential of nanoparticles as carriers for anticancer drugs for the treatment of cancers, including solid tumours.^37–39^ Some of these nanoparticles have been approved, while some are in preclinical and clinical trials for various cancers, including solid malignancies, breast, ovarian and lymphoma.^40,41^ Their unique size is optimal for an enhanced permeability and retention effect in the tumour microenvironment. They are large enough not to leak through the vasculature and small enough to be cleared from circulation by phagocytosis. Nanoparticle delivery systems can overcome physiological barriers and deliver bioactive compounds to the target site. Furthermore, they are employed in actively targeting the tumour by surface modification with molecules that bind with overexpressed receptors or antigens.^42^ Nanoparticles in combination therapy have been shown to help combat multidrug resistance in different cancers.^43–45^ In addition, nanoparticles are employed in cancer theranostics, an approach where anticancer and imaging agents are combined in a single system.^46–48^

In this review, conventional and emerging therapies for retinoblastoma were discussed, with an emphasis on the recent developments, challenges and prospects of the use of nanoparticles as novel delivery systems for chemotherapeutic drugs and imaging agents. The potential use of nanoparticles in active drug targeting and in the delivery of phytochemicals to the retinoblastoma cells was also discussed.

## 2. Treatment options for retinoblastoma

### 2.1. Current treatment options

Depending on the staging, retinoblastoma can be treated by surgery (enucleation), radiotherapy, cryotherapy, laser therapy (thermotherapy or photocoagulation), chemotherapy (systemic or local) or a combination of these. These therapies, their indications and side effects are summarized in Table 1.

**Table 1:** Current treatment options of retinoblastoma, their mechanisms, indications and side effects

Therapy		Mechanism of action	Indications	Side effects
Radiotherapy	Brachytherapy (plaque radiotherapy)	Use of high-energy radiation from a plague placed on the sclera of the eye	Solitary (or at most two foci), medium-sized tumours (6–15 mm) located away from the fovea and optic nerve	Risk of secondary cancer, eye dryness, cataract, retinopathy, glaucoma, optic neuropathy, scleral necrosis, strabismus, and skin reaction
			Tumour consolidation following chemoreduction	
			Residual or recurrent small-volume, active retinoblastoma	
	External beam radiation therapy	High-energy radiation from an external source	Multifocal large tumours unresponsive to focal therapies and chemotherapy	High risk of secondary cancer, cataract, dry eye, foggy vision, corneal vascularization, bony orbital abnormalities, skin reaction
			Tumours close to the macula or optic nerve	
			Retinoblastoma with vitreous seeding	
			As consolidation therapy after systemic therapy of metastatic tumour	
Cryotherapy		Freezing of tumour cells using a nitrogen oxide probe	Small (<3.5 mm base and <2 mm thickness) tumours anterior to the equator. Tumour consolidation following chemoreduction for more advanced tumours	Damage to the retina, including retinal tears, retinal detachment, retinal fibrosis, proliferative vitreoretinopathy, and chorioretinal atrophy
Laser therapy	Photocoagulation	Use of a laser beam to heat up and destroy blood vessels around the tumour	Small (<4.5 mm at the base and <2.5 mm thick) posterior tumours	Vascular occlusions, retinal traction, and retinal detachment
			Tumour consolidation post chemoreduction	
			Tumour-associated retinal neovascularization	
	Thermotherapy (transpupillary thermal therapy)	Use of infrared light to direct sub-photocoagulation heat to the tumour	Small (<3 mm) tumours posterior to the equator	Retinal fibrosis, retinal traction, retinal detachment, and vascular occlusion
			As a consolidation therapy after chemoreduction	
Chemotherapy	Systemic chemotherapy	Administration of cytotoxic drugs into the systemic circulation	To reduce tumour volume and to increase the effectiveness of focal treatments (chemoreduction)	Neurotoxicity, ototoxicity, bone marrow suppression, nephrotoxicity, presence of ocular blood barriers
			As an adjuvant treatment to prevent metastasis following enucleation	
			Metastatic retinoblastoma	
			To reduce the long-term risk of secondary cancers	
	Intra-arterial chemotherapy	Administration of anticancer drugs directly into the eye through the ophthalmic artery	For advanced stage retinoblastoma	Haemorrhage, stroke, loss of limb, vision loss, death
			Recurrent tumours following previous systemic chemotherapy or plaque radiotherapy	
			Recurrent subretinal and vitreous seeds	
	Intravitreal chemotherapy	Administration of cytotoxic drugs directly into the vitreous cavity through the pars plana	Subretinal and vitreous seeds of tumour unresponsive to other treatments	Transient vitreous haemorrhage, chorioretinal atrophy, and extraocular tumour spread
			Recurrent or residual vitreous seeds	
Enucleation		Surgical removal of the intact eye	Advanced stage retinoblastoma	Vision loss, facial deformity
			Retinoblastomas that are unresponsive to conservative therapies	
			To prevent metastasis	
			Presences of neovascular glaucoma, vitreous haemorrhage, cataract, corneal opacity	

#### 2.1.1. Radiotherapy

Brachytherapy (plaque radiotherapy) and external beam radiation therapy (EBRT) are two types of radiation therapy used in retinoblastoma. Both therapies employ high-energy radiation to kill tumour cells but differ in the location of the radiation source. For brachytherapy, a plague containing a radioactive agent is placed on the sclera (the outer part of the eye) to deliver the radiation. Brachytherapy is used for small-to-medium-sized tumours located more than 3 mm from the optic disc. External beam radiotherapy uses radiation from a source outside the body. EBRT can be used to treat retinoblastoma that is not responsive to other treatment options and for tumours close to the optic nerve. It is only used when necessary because of its associated risks, including second cancer risk in patients with heritable retinoblastoma, secondary sarcoma, retinopathy, midface hypoplasia and cataract.^49,50^ After radiation therapy, enucleation will still be needed owing to radiation-induced complications.

#### 2.1.2. Cryotherapy

Cryotherapy involves using a probe placed on the sclera to deliver liquid nitrogen to adjacent tumours, freezing and killing the cells.^51^ It is used for small tumours in the anterior part of the eye. The possible side effects of cryotherapy are retinal detachment, vitreoretinopathy and chorioretinal atrophy.^7^

#### 2.1.3. Laser therapy

Laser therapy of retinoblastoma can be achieved through photocoagulation or thermotherapy. Photocoagulation uses a laser beam to heat up and destroy blood vessels around the tumour. This therapy is effective for small tumours located at the posterior eye away from the optic disc and fovea. Complications of this treatment include vascular occlusions, retinal traction, and retinal detachment.^52,53^ Thermotherapy (transpupillary thermal therapy) uses infrared light directed to the tumour to kill the cancer cells. This treatment option can be used only for small tumours, even those adjacent to the fovea or optic disc. It can be combined with chemotherapy or radiotherapy for large tumours.^54^ Complications of thermotherapy include cataracts, retinal fibrosis and iris atrophy.

#### 2.1.4. Chemotherapy

Chemotherapy utilizes cytotoxic drugs to kill cancer cells. Chemotherapeutic agents can be administered systemically (intravenous) or locally (intra-arterial or intravitreal). Commonly used anticancer drugs are carboplatin, cisplatin, etoposide, vincristine, topotecan and melphalan. Systemic chemotherapy (chemoreduction), using a combination of two or three anticancer drugs, is employed to shrink tumours and increase the effectiveness of focal treatments (laser therapy, cryotherapy or brachytherapy). Adjuvant and intrathecal chemotherapies are utilized when cancer has metastasized to other parts of the eye and body, even after surgery. However, systemic chemotherapy is associated with severe side effects like secondary cancer, transient neutropenia, hyponatremia, and damage to the liver, ear and kidney.^55,56^ These effects arise mainly due to their non-specific absorption and distribution throughout the body. Systemic chemotherapy is usually followed by consolidation with focal therapy.

Local chemotherapy is a novel approach developed to overcome the drawbacks of systemic chemotherapy. It involves the injection of anticancer drugs directly through the ophthalmic artery or into the vitreous cavity of the eye, thereby increasing drug concentration at the target size and reducing systemic side effects. Vitreous seeding, which occurs at the advanced stage of cancer, is effectively controlled by intravitreal chemotherapy. This local chemotherapy has tremendously helped in salvaging the eye in patients with advanced retinoblastoma.^15,16^ Local chemotherapy with melphalan has been reported to cause vitreous haemorrhage, retinal pigment epithelial alterations, pulmonary toxicity, arrhythmias and retinal vasculitis.^18,19^ Local chemotherapy is expensive, requires skilled personnel, and is not accessible to patients in developing countries. Therefore, surgery is still the mainstay therapy for advanced retinoblastoma in those countries.

#### 2.1.5. Enucleation

Enucleation, the surgical removal of the eye, is the fastest and least costly treatment, reduces mortality and has no systemic complications.^57^ Its non-conservative nature results in permanent loss of the eye and facial deformity. Thus, it is still reserved for the very advanced stage of retinoblastoma.

**Fig. 2:**
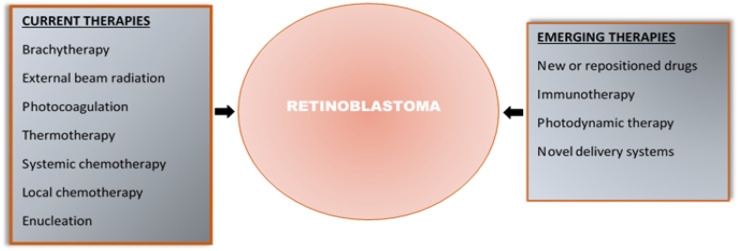
Current and emerging therapies for retinoblastoma.

Fig. 2 summarises the current and emerging therapies for retinoblastoma.

### 2.2. Emerging treatment options for targeting retinoblastoma

Emerging treatment options are dedicated research efforts geared towards overcoming the shortcomings of the current treatment regimens. Therefore, it is pertinent to overview the current treatment options for retinoblastoma and their shortcomings, which provides the bases for dedicated research efforts in the development of novel, repositioned therapeutic agents or delivery systems that will improve clinical efficacy and greatly reduce toxicity, thereby improving the prognosis of retinoblastoma.

#### 2.2.1. New or repositioned drugs

**Fig. 3:**
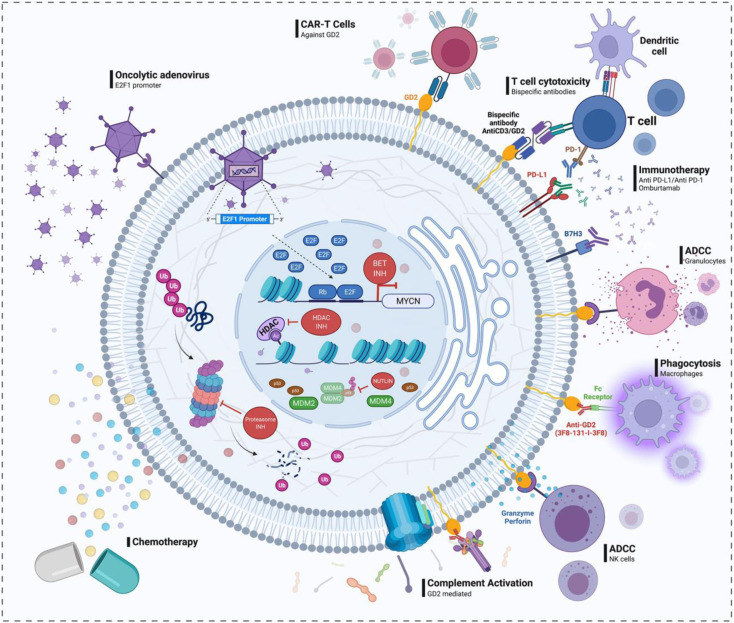
Pathways for new drug discovery in retinoblastoma. Reprinted with permission from ref. 58. Copyright 2022, Frontiers in Oncology 2022, Pharmaceutics.

The side effects of conventional drugs, such as melphalan and etoposide, include organ damage and a high risk for the development of secondary tumours. The high incidence of adverse effects with these drugs has increasingly necessitated the search for novel or repositioned drugs with intrinsic selective action on the ocular tissue tumour cells. Current approaches for the development of ocular-specific novel drug candidates for the treatment of retinoblastoma are through new drug discovery and repurposing of old drugs. Drug discovery is achieved using innovative technology to identify deregulated pathways in the development of retinoblastoma. Drug repositioning is a faster and cheaper approach and involves using old drugs for new therapeutic indications.^58^ To this end, one high throughput screening study identified cardenolides as active against retinoblastoma.^59^ In this study, a patient reportedly got cured of retinoblastoma after an ophthalmic artery chemosurgery delivery of digoxin; however, a cataract developed as a consequence, probably as a result of the route of administration.^59^ Optimizing the route of administration of this repurposed drug, digoxin, might reduce its cardio and ocular toxicities. Another approach is identifying tumour targets and developing direct treatments.^59^ To this end, inhibitors of Bcl-2 proteins, the proteasome, bromodomain and extra-motif proteins (BET), NF-KB, histone deacetylase, kinesin spindle protein, STAT 3 and survivin have been identified (see Fig. 3) and are in the early stages of drug development.^59^ Results from genomic and transcriptomic analyses of retinoblastoma tumours have shown the deregulation of DNA repair proteins, RAD51 and BRCA1. A synergistic antitumor effect was demonstrated by a combination of the chemotherapeutic drug topotecan and a specific inhibitor of RAD51.^60^ The most investigated deregulated pathway in retinoblastoma is the MDM2/MDM4-p53 pathway (see Fig. 3).^61^ In retinoblastoma, the overexpression of MDM2-p-53 and its homologue MDM4-p-53 blocks the transcription process and triggers the degradation of the P-53 protein.^62^ Phase I and II clinical trials on the use of MDM2/MDM4 inhibitors are ongoing.^58^ Another emerging option is targeting HDM2, another p53-negative regulator shown to cause death in retinoblastoma cells.^63^ Other pathways of drug discovery include the MYC/MAX signalling, which has prompted the search for MYC/MAX inhibitors, BET inhibitors and the repurposing of calcium and potassium channel blockers in this regard.^64^

#### 2.2.2. Targeted drug delivery

Targeted drug delivery has become pertinent due to systemic and local toxicities associated with administering conventional chemotherapeutic agents in the treatment of retinoblastoma. Targeted drug delivery of chemotherapeutic agents concentrates the drug on the desired site of action, thus reducing the side effects of the drug while improving the efficacy of the drug and the prognosis of treatment. Furthermore, targeted delivery of chemotherapeutic agents is of great essence due to the low bioavailability of drugs in the posterior segment of the eye leading to high treatment failure rates, among other setbacks.^65^ Retinoblastoma cells overexpress some receptors which exhibit affinity for several ligands such as galactose, mannose, folic acid and hyaluronic acid, thus providing a means for retinoblastoma cell-targeted drug delivery.^66^ Active drug targeting to retinoblastoma using nanoparticles is discussed in detail in Section 5.

#### 2.2.3. Immunotherapy

Immunotherapy holds a lot of promise in the development of targeted and precise treatments for retinoblastoma. In retinoblastoma cells, the ganglioside GD2 and neural cell adhesion glycoprotein CD171 are highly expressed on their surfaces and are targets for recently designed CAR T cells. These CAR T cells, when administered to a panel of retinoblastoma cells, were shown to be cytotoxic.^67^ Subsequently, the anti-GD2 CAR T cell-based formulations developed for intravitreal administration in the treatment of retinoblastoma gave a complete anti-tumour response and were shown to cause no recurrence or ocular toxicity.^68^

#### 2.2.4. Novel drug delivery systems

Novel drug systems such as hydrogel, liposome, polymeric, lipid and inorganic nanoparticles have been investigated for delivering chemotherapeutic agents to retinoblastoma cells.^69,70^ They have shown excellent potential for targeted, controlled and sustained delivery while minimizing the adverse effects. These systems act as carriers not just for cytotoxic drugs but also for genes and imaging agents. They can also be surface-modified or functionalized to improve their pharmacokinetic profiles. The advantages of novel drug delivery systems over conventional treatment therapies for retinoblastoma are listed in Table 2.

**Table 2:** Advantages of novel delivery systems over conventional treatment options

Advantages of novel drug delivery systems in cancer therapy
Enhanced solubility and stability of anticancer agents
Co-delivery of two or more anticancer agents
Long circulation half-life
Passive targeting enhancing permeation and retention effects
Ability to navigate the tumour microenvironment and prevent multidrug resistance
Active targeting increasing cellular uptake and internalization
Low systemic/off-target toxicity
Controlled drug release
More specific and sensitive imaging
Better imaging contrast enhancement
Safer MRI contrast agents
Multimodality imaging
Theranostic potential (ability to image and treat simultaneously)

## 3. Nanoparticles as an emerging treatment option for retinoblastoma

**Fig. 4:**
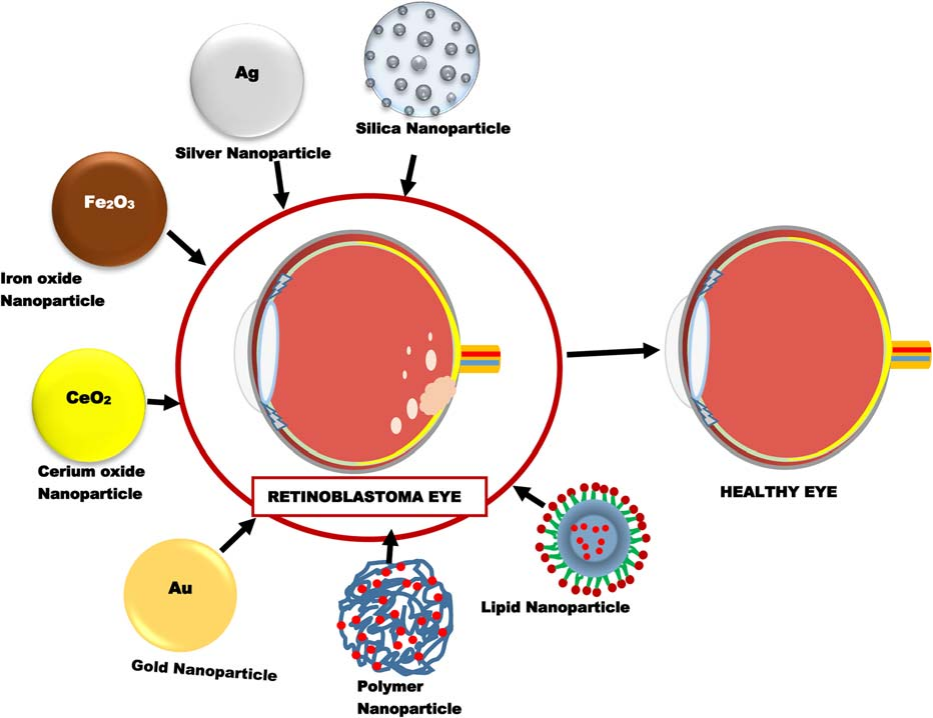
Different types of nanoparticles used in retinoblastoma therapy.

Nanoparticles are one of the most extensively investigated novel drug delivery systems for retinoblastoma therapy. Different types of nanoparticles that have been recently studied for use in retinoblastoma include inorganic, lipid and polymeric nanoparticles (Fig. 4).

### 3.1. Gold nanoparticles

Gold nanoparticles (GNPs) are usually seen as brilliant red with increased surface area.^71^ The chemistry of the GNPs enables their surfaces to be tailored to receive or carry desired charges, be made hydrophilic, and can be functionalized for targeting. GNPs have peculiar features, such as the propensity to take in near-infrared light, which allows for the photothermal annihilation of malignant cells.^72^ They are not efficient fluorophores as their fluorescence quantum is about 10^−5^ or less, transforming the light absorbed by the nanoparticles into heat. This behaviour has some advantages, such as controlled release behaviour.^73^ Yavuz *et al.* reported a similar case where the encapsulation of gold nanocages was carried out with a smart polymer loaded with doxorubicin.^74^ The absorption of light by the nanocages results in their conversion to heat. This, in turn, causes the breakdown of the polymer and the subsequent release of doxorubicin. The light responsiveness of GNPs further buttresses their potential for use in ophthalmic diseases such as retinoblastoma.^74^ In another study, brachytherapy (internal radiation) performed by the technique of ultrasonic hyperthermia together with GNPs was studied on a retinoblastoma tumour injected into an animal model (rabbit). Findings show that a mix of the above therapy resulted in the depletion of the relative size of the tumour. Subsequently, an increase in the percentage of cell death and a significant reduction in the retinoblastoma mass in the rabbit's eyes was reported.^75^ The GNPs have also found application in theranostics, a platform for imaging and therapeutics. This was demonstrated when mesoporous gold nanocages (AuNCs) were conjugated with Fe_3_O_4_ nanoparticles, and muramyl dipeptide (MDP), an immunomodulator, and perfluorobutane (PFP) were encapsulated. This formulation was intended for retinoblastoma diagnostic imaging and targeted therapy. Profound cytotoxicity was reported in retinal pigment epithelial ARPE-19 cells and retinoblastoma Y79 cell lines. In addition, *in vivo* studies in mice also confirmed both diagnostic and therapeutic activity.^76^ In summary, GNPs are extremely flexible nanomaterials that can be adapted in various delivery strategies for the treatment of retinoblastoma.

### 3.2. Silver nanoparticles

Silver nanoparticles (AgNPs) refer to nanoscale-sized particles of silver with sizes typically between 1 and 100 nm. These particles have unique electrical, optical, and magnetic properties with a wide range of applications. Additionally, they have a large surface area. The AgNPs exert DNA damage and the death of malignant cells *via* the release of reactive oxygen species. There are, however, few studies on the use of AgNPs for retinoblastoma care or treatment.^77^ According to Remya *et al.*, *Turbinaria ornata* was used as a natural source for the manufacture of AgNPs, and its cytotoxic profile against retinoblastoma cells was assessed.^78^ The inhibitory concentration (IC_50_) of 10.5 g ml^−1^, which measured the cytotoxicity of the produced AGNPs against the RB Y-79 cell line, showed increased cytotoxicity with an increased dose. According to their findings, AgNPs are effective anticancer medicines with augmented ocular targeting and management.^78^ Another study found evidence that AgNPs can cause oxidative stress and cell death. In *ex vivo* grown post-natal mouse retina, the ability of retinal tissue to absorb low concentrations of nano-sized AGNPs was investigated. The retinas were tested for NP uptake after 72 hours of NP exposure. The effects of silver nanoparticles on oxidative stress and apoptosis were also assessed utilizing biochemical tests. Results indicated that AgNPs were taken up by and internalized by retinal cells and exert toxic effects on the retina, including apoptosis and oxidative stress, which suggest a direct cytotoxic effect.^79^ Although limited reports are seen on the use of AgNPs, their potential for drug loading or conjugation protocol for the treatment of retinoblastoma can still be explored.

### 3.3. Iron oxide nanoparticles

Iron oxide nanoparticles (IONPs) have drawn much attention due to their distinctive qualities, including superparamagnetism, larger surface area, and simple separation approach.^80^ IONPs have found diverse applications in various areas of sciences, including biomedical, diagnostics and drug delivery. IONPs can be induced into magnetic resonance by hyperthermia, applying an external magnetic field; therefore, travelling along the field of attraction is possible. The nanoparticles can also have organic or inorganic molecules surface-coated to increase the likelihood of effective drug delivery. These molecules include surfactants, medicines, proteins, starches, enzymes, antibodies, nucleotides, nonionic detergents, and polyelectrolytes.^81^ According to some studies, IONPs are used to treat retinoblastoma. Demirci *et al.* reported the application of various doses of IONPs, which were surface coated with dextran.^82^ A magnetic hyperthermic pattern was utilized to investigate the preferential death of Y79 retinoblastoma and ARPE-19 retinal pigment epithelium cells by treating them with dextran-coated IONPs. Tumour cells are more sensitive to heat than normal cells, thereby making hyperthermia a useful therapeutic technique in the treatment of cancer. Different methods, such as radio frequency, microwaves, and concentrated ultrasound, can raise the temperature. Iron nanoparticles have been employed as nano-heaters that can target tumour cells without harming healthy tissue through a process known as magnetic hyperthermia. At concentrations of 0.5 mg ml^−1^ and above, dextran-coated iron oxide NPs, through magnetic hyperthermia, selectively destroyed more than 70% of the Y79 cells by activating apoptotic pathways.^82^ Another study investigated the use of iron oxide nanoparticles when conjugated to gold nanocages, which were loaded with drugs as a platform for imaging and therapeutics. Results showed that diagnostic imaging and therapy were achieved when tested on retinal pigment epithelial ARPE-19 cells and retinoblastoma Y79 cell lines.^76^

### 3.4. Mesoporous silica nanoparticles

**Fig. 5:**
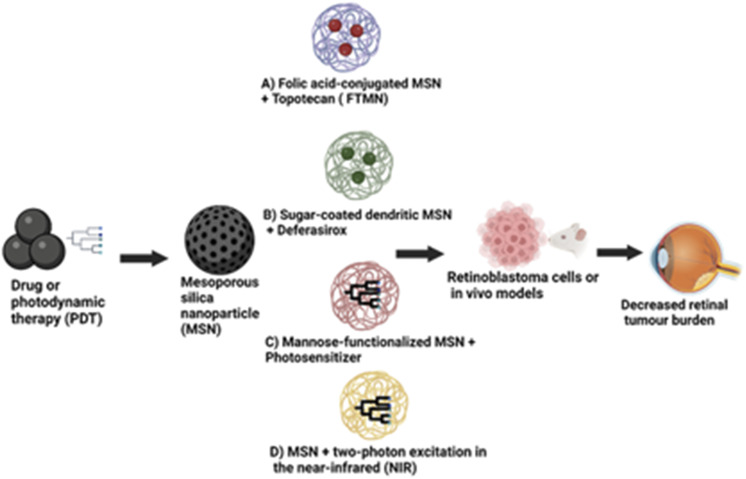
Some applications of mesoporous silica nanoparticles (MSNs) in retinoblastoma therapy.

Mesoporous silica nanoparticles (MSNs) stand out among various nanoparticle systems due to their enormous surface area, nano-sized particles, surface modifications, tunable pore size, and pore shape.^83,84^ They also exhibit exceptional colloidal stability. Due to their unique characteristics, including biocompatibility, MSNs have been investigated as a potential therapy for retinoblastoma.^84,85^ One method of employing MSNs in treating retinoblastoma entails dosing them with a chemotherapeutic agent and directing them, particularly to cancer cells in the eye. MSNs' large surface areas, variable pore sizes, and capacity to be functionalized with targeting ligands make it possible for them to load medicines efficiently and bind to cancer cells specifically, as shown in Fig. 5.In a study by Qu *et al.*,^86^ they developed folic acid-conjugated MSNs (FA-MSNs) for improved topotecan treatment efficacy in retina tumours. Their study showed that these nanoparticles loaded with topotecan (FTMNs) showed greater cellular uptake and consequently higher *in vitro* cytotoxicity against Y79 retinoblastoma cells compared to topotecan alone or non-targeted nanoparticles.^87^ The live/dead assay results and the nuclear fragmentation assay demonstrated that the FTMNs consistently induced cell apoptosis of the retinoblastoma cells with up to 58% efficacy. The *in vivo* investigation also revealed a more substantial tumour-inhibitory impact of FTMNs than other formulations or topotecan alone. As anticipated, FTMNs showed a striking decrease in the total tumour volume compared to any other group with reduced tumour cell presence in histological staining. The findings imply that FA-MSNs can improve topotecan's therapeutic effectiveness in the management of retinoblastoma.^87^ A similar study employed sugar-coated dendritic MSNs for pH-responsive tumour targeting and controlled release of the anticancer drug deferasirox. The *in vitro* drug release study showed that polyamidoamine dendrimer with sugar conjugation modified onto the MSNs served as a “gatekeeper”.^88^ This formulation blocked the pores of the MSNs, causing pH-dependent controlled drug delivery (full release at pH 4.5) compared to the rapid release observed in the MSN-only condition. Additionally, the sugar-coated MSNs demonstrated higher cytotoxicity in the Y79 retinoblastoma cell line, indicating enhanced cellular uptake of these nanoparticles in the tumour cells, which raises the possibility that they could serve as an effective nanocarrier for cancer therapy.^88^

Some studies have reported the application of MSNs in photodynamic therapy, a minimally invasive cancer treatment that kills cancer cells by exposing them to light and photosensitizers. The silicalites and MSNs exhibited high photosensitizer loading capabilities; when exposed to light, they were able to produce reactive oxygen species (ROS), which had a strong lethal effect on cancer cells.^89–92^ Due to their greater surface area and pore capacity, the MSNs produced ROS more efficiently than their silicalite counterparts.^91^ Mannose-functionalized MSNs of 100 nm diameter were employed to target retinoblastoma cells through active endocytosis by mannose receptors. These modified MSNs were effective for retinoblastoma cell imaging, drug administration, and photodynamic treatment. This finding suggests that the use of MSNs may provide various benefits over conventional PDT agents, including superior photosensitizer targeting and controlled release, as well as increased efficacy because of their unique features. However, their significant diameter limited further use. Additional research from this group showed that smaller-sized mannose-functionalized MSNs (25 nm) could target and image retinoblastoma cells.^90^ Confocal imaging of retinoblastoma cells was performed using a stable suspension of MSNs in phosphate buffered saline. Fast endocytosis of the MSNs was seen within 5 hours. The results confirmed that the MSNs were biocompatible. They demonstrated increased cellular uptake and fluorescence signal compared to the non-functionalized MSNs.^90^ C-type mannose receptor 2 (MRC2) and CD209 receptors are highly expressed in the retinoblastoma and were targeted using MSNs loaded with anti-MRC2 and anti-CD209 antibodies in photodynamic therapy and retinoblastoma cell imaging.^92^

Similarly, two-photon excitation therapy can also be applied to treat retinal tumours. Silica and organosilica nanoparticles, activated by two-photon excitation in the near-infrared (NIR), allowed for the exact and selective targeting of cancer cells in deep tissues. Following exposure to NIR light, the nanoparticles selectively aggregated in the tumour tissue and caused tumour regression.^93^ Additionally, MSNs have been used to treat other cancer types, including localized breast tumours with doxorubicin-loaded nanofibers, and to increase the drug accumulation of doxorubicin in resistant breast cancer cell lines.^94,95^ Overall, MSNs have great potential for treating retinoblastoma. Additional research is required to enhance the drug delivery design and ensure their safety and efficacy in human clinical trials across various malignancies to provide a flexible platform for targeted cancer therapy.^90,92,93^

### 3.5. Cerium oxide nanoparticles

Cerium oxide nanoparticles (CNPs), or nanoceria, comprise cerium and oxygen atoms. They stand out due to their exceptional self-generating antioxidant capabilities, high surface area-to-volume ratio, and strong catalytic activity.^96,97^ Depending on the surrounding conditions, nanoceria has the remarkable capacity to transition between the (+3) and (+4) oxidation states, making it the most critical rare earth metal oxide. Due to the redox behaviour of cerium, it can exist as CeO_2_ and Ce_2_O_3_ in the bulk state and exhibits catalytic activity.^98^ It possesses a highly reactive surface area for neutralizing free radicals because it can assume a fluorite crystalline lattice structure. Because of the formation of oxygen vacancies in the lattice structure of nanoceria as its size decreases, these oxygen defects serve as a free radical scavenger in the physiological environment.^97^ These characteristics have attracted much interest in many biomedical applications, including drug administration, imaging, and therapy.^99^ Some studies have focused on nanoceria's *in vitro* and *in vivo* applications in retinoblastoma therapy.^100–103^

Gao *et al.* investigated using a pH-responsive nanoceria system for targeting retinoblastoma.^100^ They developed an anti-tumour delivery system that combined extracellular pH-dependent functionality, a tumour cell targetable (CXC chemokine receptor 4, CXCR4 receptor specific) antagonist (AMD11070), reactive oxygen species inducible glycol chitosan-coated nanoparticles and the anticancer drug doxorubicin. The integrated nanoparticle system was studied in WERI-Rb-1 and Y79/GFP-luc-induced genetic p107s mice and retinoblastoma cells. For their efficacy studies, the nanoparticles demonstrated significant inhibition of tumour cell growth at pH 6.5 (tumour acidic environment) and avoidance of retinal blood vessel leakages. Also, the integrity of the retina and lens of the mouse model was protected. The smart nanoparticle with a neutral surface charge at physiological pH was hypothesized to promote diffusion through the vitreous matrix to the retina tumour site and induce an anti-tumour effect by accumulating a positive charge.^100^ The system's sensitivity to pH enables targeted drug release in the tumour microenvironment, reducing side effects and enhancing treatment effectiveness. The sol–gel technique has also been used to synthesize cerium-doped titania nanoparticles, which showed improved photodynamic anticancer effects in Y79 retinoblastoma cells. Kartha *et al.* synthesized and characterized these Ce-doped TiO_2_ nanoparticles using different analytical techniques, including transmission electron microscopy, X-ray diffraction, and Fourier-transform infrared spectroscopy.^101^ They proposed that Ce-doping boosted the surface area of TiO_2_ nanoparticles and improved their photocatalytic activity compared to the undoped nanoparticles, both promoting cancer cell death.

Additionally, they discovered that the Ce-doped TiO_2_ nanoparticles caused cell cycle arrest and apoptosis in the retinal cancer cells.^101^ Mouse model (P53TKO mice, Chx10-cre; Rb Lox/−; p53 Lox/−; P107−/−) studies on the role of CNPs on ocular tumours showed a greater than 50% decrease in the tumour size at about three weeks following a single dose of intravitreal injection of these nanoparticles.^102^ These findings suggest that nanoceria are potent tumour growth inhibitors and may be a novel therapeutic strategy for ocular malignancy.

### 3.6. Lipid nanoparticles

**Fig. 6:**
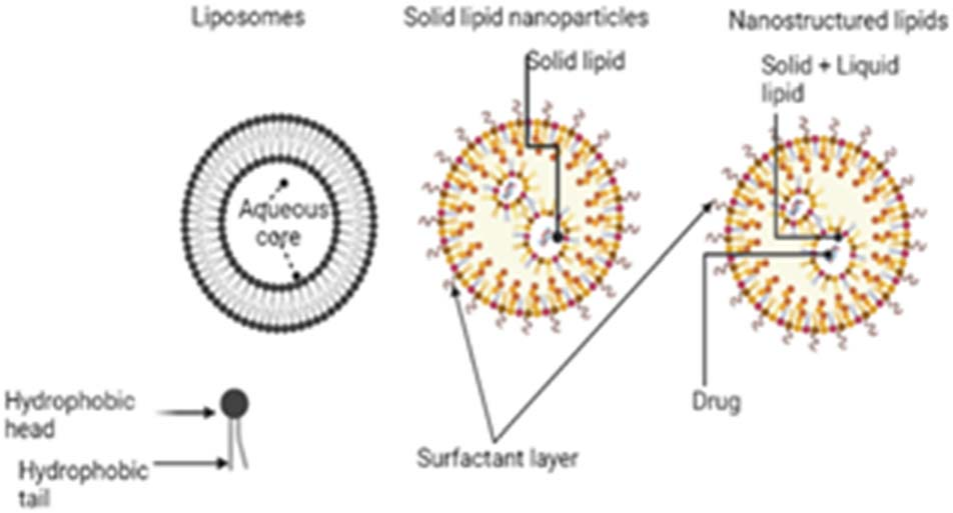
Lipid nanoparticles in retinoblastoma; the figure was created with https://www.BioRender.com.

Nanoparticles based on lipids have lipid molecules serving as the structural backbone of the particles. Because of their biocompatibility and versatility in ocular delivery, lipid-based nanocarriers have emerged as promising alternatives for delivering therapeutic drugs to the targeted cells in the eye. Lipid nanoparticles (LNPs) have been employed in nucleic acid delivery technology to treat ocular illnesses since they can pass the ocular barrier and efficiently transfect nucleic acids to various cells of the eye. The size of lipid nanocarriers determines their cellular absorption, diffusion, and biodistribution, making them an attractive platform for targeted drug administration.^104^ Lipid-based nanoparticles include liposomes, solid lipid nanoparticles (SLNs) and nanostructured lipid carriers (NLCs). The structures of these lipid carriers are shown in Fig. 6.

The low cellular and systemic toxicity of the lipids enhances their biocompatibility profile. SLNs protect drugs from chemical and enzymatic breakdown and increase bioavailability.^105^ They are lipid biocompatible and can be delivered by all routes. The solid nature of the lipids employed in SLNs at room and body temperature ensures stability, controlled drug release, and target delivery. Because of the disorganized core made up of both solid and liquid lipids, NLCs can load more drugs than SLNs. Liposomes and solid lipid nanoparticles (SLNs) are very effective at delivering gene segments to target cells while simultaneously allowing for the regulated release of the drug in both *in vitro* and *in vivo* settings. Liposomes and SLNs can have cationic surface charges, which makes it easier for anionic gene segments to bind to the lipid structure of the liposomes and SLNs.^39,106,107^ Because clathrin is often required for their absorption, they are particularly amenable to having their structures altered in order to improve their capacity for transfection or their ability to avoid early endosomal degradation.^108,109^ A low concentration of cationic charge within the cell ensures that the drugs will be released with maximum efficiency. It is possible for reporter genes delivered *via* subretinal injection of SLNs to effectively infiltrate retinal pigment epithelium (RPE) and photoreceptor cells.^110^ In order to ensure that optimum delivery and therapy are provided, Tabatabaei *et al.* produced 171 nm switchable LNPs for the codelivery of melphalan and miR-181.^39^ Ethanol was used to enhance the loading of the antitumor agents in the nanoparticles. The encapsulation efficiency of miR-181 was determined to be 93% by fluorescence displacement assay. LNPs boosted the expression of apoptotic genes, and the results showed that they had the maximum absorption and targeted death of retinoblastoma cells. Several characterization approaches were used to evaluate formed nanoparticles, and the findings revealed that LNPs are the most effective.^39^ Ahmad *et al.* also synthesized SLNs to deliver etoposide in a manner that is both safe and targeted against retinoblastoma.^111^ Melt-emulsification and ultrasonication were the two methods that were utilized in the SLN synthesis process. The SLNs were analyzed to determine their size, surface morphology, entrapment efficiency, and *in vitro* drug release. However, pharmacokinetic experiments were performed on Wister rats following the intravitreal administration of the SLN formulation. In addition, an albino rabbit's ocular tissues were subjected to a gamma scintigraphic examination to determine whether SLNs had been deposited there. In gamma scintigraphy, radioisotopes are injected into the bloodstream. These radioisotopes are designed to keenly find out bone that has been irritated, damaged, or repaired, as well as inflammatory or necrotic tissues. After that, histological investigations are carried out to evaluate the toxicity and the morphological changes that occurred as a result of the treatment. In spite of this, it was deduced from the findings that the optimized formulation had a particle size of 239.43 nm, a PDI of 0.261 ± 0.001, and an EE of 80.96% and 2.21%, respectively. The most interesting aspect of this formulation was that it allowed for prolonged drug release over seven days with only a single intravitreal injection. The findings of the gamma scintigraphy investigation corroborated the idea that the drug release was maintained for a period of seven days. Histological studies proved that SLNs are not harmful since adverse effects were not observed in the posterior eye tissues of examined animals. Thus, etoposide-loaded SLNs are both effective and secure in the treatment of retinoblastoma.^111,112^ In another study conducted by Marathe *et al.*, the authors employed a paclitaxel-loaded, α-tocopherol succinate-based nanostructured lipid carrier to increase the bioavailability of the drug in the eye.^113^ The formulations demonstrated good physicochemical features and could lead to a successful therapeutic alternative in the management of retinoblastoma if more research on them is conducted.^113^

### 3.7. Polymeric nanoparticles

Polymeric nanoparticles (PNPs) are one of the most useful multi-functionalized nanoparticles in the treatment of retinoblastoma.^112^ They have been widely studied and have received enormous attention compared to other nanoparticles. Hence, PNPs can be more effective in targeting and killing cancerous cells.

A study used polylactic acid nanoparticles encapsulating rhodamine dye in a rat model for retina delivery.^114^ Following a single intravitreal injection, the nanoparticle could facilitate its diffusion through the retinal layers and concentrate in the retinal pigment endothelium for up to 4 weeks. However, in the posterior vitreous humour as well as the ciliary body, there was a mild inflammation which reduced after 48 h.

In another study, a micellar delivery system consisting of the biodegradable poly(lactic-*co*-glycolic acid) polymer and hydrophilic polyethylene glycol was evaluated. This system was used to deliver doxorubicin to retinoblastoma cells in a sustained release system for 2 weeks *via* the intravitreal route.^115^ The micelles were prepared using dimethylsulfoxide, acetone or dimethylformamide solvents. The solvent effects on the efficiency of entrapment, size of the particle, polydispersity and the effect of the gel structure on the release of DOX were also investigated. The result indicates that dimethylformamide was the best solvent for the micelle preparation and that the dispersion of doxorubicin in PLGA–PEG–PLGA gel produced a sustained drug release for up to 2 weeks. The rate of doxorubicin uptake was higher with doxorubicin micelles than free doxorubicin in Y-79 cells with excessive folate expression. The result suggests that polymeric micellar systems, which are suspended in gels, are thermosensitive and may provide targeted delivery of anticancer chemotherapeutics.

A drug delivery system consisting of polymers like poly(lactic-*co*-glycolic acid) and polycaprolactone, which are noted for their biocompatibility and distinct degradation rates, blended with polymeric nanoparticles as an effective chemotherapeutic agent was highlighted and presented.^116^ Palbociclib, an anticancer drug approved by the FDA, and a near-infrared dye, IT820 (IR), were entrapped as chemo/phototherapeutic agents in multifunctional polymer nanoparticles encompassing the hydrophobic moieties and synthesized by the method of solvent emulsification. The hydrophobic entities were entrapped effectively in the hybrid polymer nanosystem, as shown by the UV-vis spectra, with the PNPs exhibiting characteristic peaks at 365 and 825 nm. The subsequent evaluation of the effect in the retinoblastoma cell line showed that the PCB/IR PNPs enhanced the cytotoxic killing effect (86.5 ± 2.3%) in Y79 cell lines than the control group upon NIR light exposure. Furthermore, to ascertain the mechanisms of PCB/IR PNP induced cytotoxic activities in retinoblastoma cell lines Y79 when exposed to NIR light, the cell lines were investigated for the possibility of DNA injury by DAPI staining, revealing that PCR/IR PNPs, in addition to NIR light treated cells, had a condensed nucleus, a typical characteristic of the process of apoptosis. However, this phenomenon was not observed in control cells. The effectiveness of PCB/IR PNPs was also tested in mice models *in vivo* and presented optimum photoacoustic signals suggesting that the blend of PCB and chemo/photothermal therapy may be a practical approach for retinoblastoma therapy.

Overall, these findings highlight the relevance of polymer nanoparticles to designing therapeutic approaches in solid tumour treatment of retinoblastoma.

## 4. Nanoparticles in delivering phytochemicals to retinoblastoma cells

**Fig. 7:**
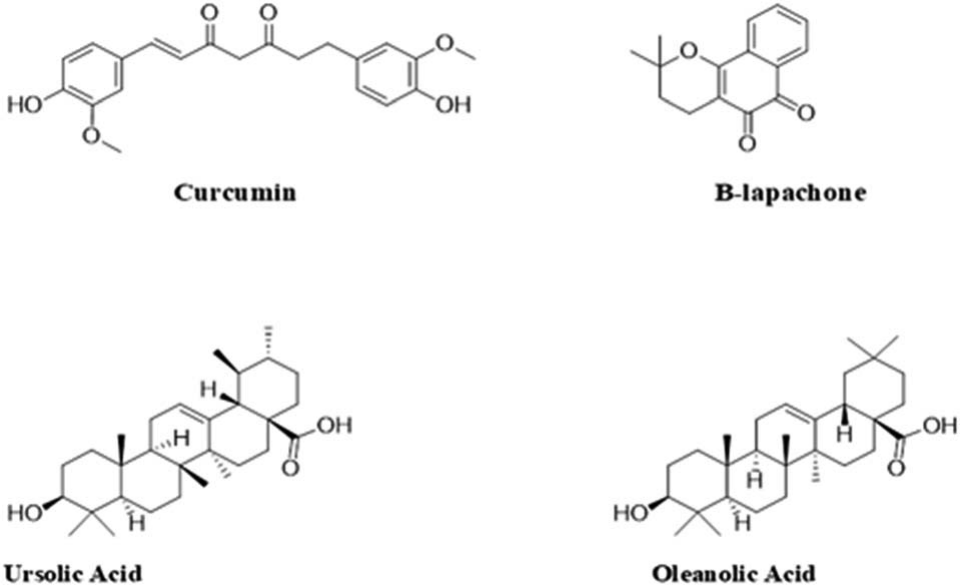
Structures of phytoconstituents investigated for anti-retinoblastoma activity.

Plants are composed of compounds with very high amounts of powerful antioxidants, such as phenolic acids, flavonoids, and carotenoids. These substances have been reported to possess activities that delay or prevent the initiation of free radicals by inactivating or scavenging these compounds, thus preventing the activation or spread of reactions *via* peroxyl or alkoxyl radicals.^117,118^ As a result, these substances, which also include sterol derivatives (*e.g.* oleanolic and ursolic acid), catechol derivatives (*e.g.* curcumin) and naphthoquinones (*e.g.* β-lapachone), have been explored over the years in the treatment of various types of cancers including retinoblastoma (Fig. 7).^85^ Studies have also reported these compounds to have fewer toxic effects than conventional chemotherapeutic agents.^85^ However, despite the benefits that these compounds possess, certain properties, such as limited solubility and permeability, have greatly impeded their application in various treatment strategies.^119^ As a result, various nanotechnological strategies have been explored to improve these limitations and consequently improve the efficacy of these compounds.

The development of phytoconstituent-based nano-formulations has received a lot of attention over the years because it combines the benefit of employing potentially less toxic phytochemicals and the advantages nano-based drug delivery systems offer.^118^ For instance, curcumin, a bright-yellow catechol-based compound produced in *Curcuma longa*, has been reported to possess diverse pharmacological effects, including anticancer effects, hence its application in the treatment of retinoblastoma.^85^ Although it is quite insoluble, several nanotechnological strategies have been employed in tackling this challenge in order to improve its use in treating various diseases. Alsaab *et al.* developed an innovative anticancer tool comprising curcumin-difluorinated loaded polymeric micelles that targeted retinoblastoma cells' folate receptors.^120^ The study showed that the micelles showed high encapsulation efficiency (>85%) and were also able to increase the solubility of the curcumin. The study also revealed that the micelles had no adverse effect on the human retinal pigment cells (ARPE-19) used in the experiments, but significantly killed the retinoblastoma cell lines (both Y-79 and WERI-RB). This suggests that the micelles were safe for non-carcinogenic cells and also very effective in eliminating cancer cells.^120^ N'Diaye *et al.* designed biodegradable lipid nanoparticles (LNPs) made of poly(d,l)-lactide (PDLLA) nanoparticles coated with a phospholipid (1-palmitoyl-2-oleoyl-*sn*-glycero-3-phosphocholine/1,2-dioleoyl-3-trimethylammonium-propane) bilayer.^121^ The nanoparticles were loaded with a phytochemical: beta-lapachone (β-Lap) (an anticarcinogenic agent) and temoporfin (a photosensitizer) to explore the benefits of combining chemo- and photodynamic approaches in the treatment of retinoblastoma. The study produced highly uniform nanoparticles with a particle size of 170 ± 3 nm and a polydispersity index (PDI) of 0.08 ± 0.02. The results from the cytotoxic studies showed that the nanoparticles were active in both chemotherapy and photodynamic therapy and could be administered in a single intravitreal injection.^121^ In a recent study, an extract of *Moringa oleifera* and a photosensitizer (IR820) encapsulated in biodegradable polymeric nanoparticles showed excellent antitumor activity when combined with photothermal therapy.^122^ The mechanism of the antitumor action was *via* the downregulation of heat shock proteins (HSP70), as reported by the authors. Hence, this combination treatment modality could prevent tumour cells from developing heat shock resistance to photothermal therapy.

Silva *et al.* developed novel oleanolic and ursolic acid-loaded polymeric nanoparticles with PLGA.^123^ These particles were negatively charged (zeta potential −27.12 ± 0.27 mV) with a particle size of 213.55 ± 1.60 nm and polydispersity index of 0.090 ± 0.038. The study produced very stable nanoparticles with significant cytotoxic activity against the Y-79 cell line. In addition to providing bioactive compounds for the treatment of RB, plants are also very effective systems for the green synthesis of cytotoxic materials.^123^ Remya *et al.* developed a rapid and effective method for the synthesis of cytotoxic AgNPs from the brown seaweed *Turbinaria ornata*.^124^ The study revealed that the particles produced *via* this method were negatively charged with a zeta potential of −28.7 mV and particle sizes ranging from 22 to 32 nm. The growth inhibitory properties of these particles were evaluated against retinoblastoma cell lines Y79, and it was observed that the viability of the cells decreased with an increase in the concentration of the AgNPs, with the inhibition concentration (IC_50_) recorded at 10.5 μg ml^−1^.^124^ Su *et al.* developed resveratrol-peptide nanospheres for the treatment of retinoblastoma.^119^ The nanospheres had a particle size of 214.10 ± 3.73 nm and very high encapsulation efficiency and drug loading (90.77 ± 3.51% and 9.82 ± 0.64%, respectively). The anticancer test, which was conducted using the Y-79 and S0-Rb50 cells, revealed that the nanospheres had a better antitumor effect than the unformulated resveratrol. This suggests that the nano-based system could represent an effective tool in the management of retinoblastoma.^119^

## 5. Active targeting of nanoparticle-enabled chemotherapeutics for retinoblastoma treatment

Cancer is one of the leading causes of death globally, claiming more than 4 million lives every year and negatively impacting patients' quality of life.^1^ Though chemotherapeutic agents do exist to combat cancer, there are limitations which hinder their applications for effective cancer therapy. One of the major challenges has been the non-selectivity and non-specificity of the cytotoxic drugs with poor tumour localization and, thus, wide distribution throughout the body. The systemic side effects resulting from the distribution of chemotherapeutic drugs to non-cancerous cells are enormous and have been recognized as one of the reasons why most potent anticancer compounds fail in clinical trials.^125,126^ Chemotherapeutic agents also face the challenges of navigating the complex tumour microenvironment in reaching the target cancer cells. The need for targeted delivery of anticancer drugs to specific tumour cells and not healthy cells cannot be overemphasized. Hence, there is a paradigm shift from conventional to more targeted cancer therapy.

**Fig. 8:**
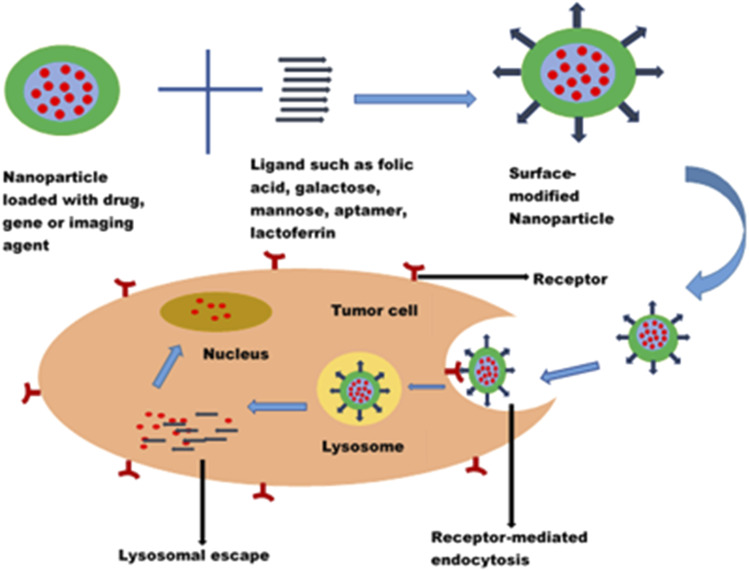
Fate of a ligand modified nanoparticle.

The advancement in the application of nanotechnology in cancer therapy stems from using surface-functionalized nanosystems to target tumour cells, thereby mitigating the systemic toxicity of chemotherapeutic drugs. Nanoparticles modified with ligands could recognize, bind, and enter the tumour cells through receptor-mediated endocytosis (Fig. 8). They exhibit an enhanced permeability and retention effect on tumour cells. In addition to reducing off-target toxicity effects, these nanocarriers can overcome the tumour microenvironment barriers, facilitating entry into cancer cells and sustained drug release. Surface modification of nanoparticles with ligands such as folic acid, galactose, lactoferrin, and EpCAM aptamer to target overexpressed biomarkers (such as folate receptors, transferrin receptors, CD44, lectins, and EpCAM) in tumour cells has shown promising results in the treatment of retinoblastoma, uveal melanoma and other ocular cancers.^112,120,127^

The most widely investigated targeting receptor in cancer therapy is the folate receptor.^120,128^ Folate receptors are expressed at high levels, around 150–300 times more in retinoblastoma and some cancers, including breast, brain, kidney and lung cancers, than in normal tissues.^129–131^ Thus, the high affinity of folic acid for folate receptors makes it an attractive targeting ligand for the preferential killing of cancerous cells.^129,132^

In a study by Das *et al.*^133^ to reverse the multidrug resistance of Y79 cells, they investigated folate-functionalized PLGA nanoparticles loaded with curcumin and a new anticancer drug, nutlin-3a. Although nutlin-3a is a potent drug that acts as a murine double minute (MDM2) antagonist and actively blocks p53-MDM2 interaction, it is a substrate for multidrug resistance proteins MRP-1 and Pgp.^134^ Hence, its clinical application is limited. Curcumin, an established modulator of multidrug resistance (MDR) proteins, could augment the antitumor activity of nutlin-3a in drug resistant Y79 cells. A targeted delivery system functionalized with folic acid was employed to increase the anticancer actions of this co-administration. The *in vitro* cellular cytotoxicity, cell cycle analysis, and apoptosis studies showed enhanced efficacy of the folic acid functionalized nanoparticles.^133^ Similarly, there was enhanced cellular uptake of topotecan-loaded mesoporous silica nanoparticles conjugated with folic acid by Y79 retinoblastoma cells.^87^

Carbohydrates are promising candidates for receptor-targeted nanocarrier drug delivery systems. The overexpression of sugar receptors (lectins) on retinoblastoma and their limited expression on healthy cells provide a distinguishing feature for the active targeting of cancer cells. Lectins are proteins which can recognize and bind specifically to sugar complexes. The lectins on human retinoblastoma cells demonstrated affinities for mannose and galactose.^66,135^ Thus, the conjugation of nanocarriers with these ligands results in selective binding with the lectins, facilitating the endocytosis of the loaded nanocarrier. Unlike folic acid, sugars do not have stability or photosensitivity concerns.^135^

Rutika Godse *et al.* developed etoposide-loaded poly(lactide-*co*-glycolide) (PLGA) nanoparticles (NPs) coated with a galactose–chitosan conjugate for preferentially targeting sugar receptors on cancer cells.^66^ A higher cellular uptake was observed with the galactose–chitosan functionalized PLGA nanoparticles (70%) than the non-functionalized nanoparticles. In addition, the loaded formulation demonstrated augmented cytotoxic and apoptosis effects compared to the pure drug.

Photodynamic therapy, as an emerging treatment option for retinoblastoma, employs irradiation of photosensitizers to generate cytotoxic ROS. It has been combined with chemotherapy, radiotherapy and other conventional treatment options for cancer diagnosis and therapy.^136,137^ Combining camptothecin delivery and photodynamic therapy using mannose- or galactose-functionalized mesoporous silica nanoparticles demonstrated approximately two times Y-29 cell death compared to the non-functionalized nanoparticles.^89^ This improved activity was attributed to the receptor-based endocytosis of the nanoparticles by the cancer cells. A synergistic therapeutic interaction was observed in the combination of photodynamic and drug therapies.

Just like carbohydrates, proteins and peptides are employed as targeting ligands. Lactoferrin (Lf) is a high molecular weight protein found in milk and a smaller percentage in bile and tears.^138^ It belongs to the transferrin family of iron-transporting glycoprotein.^128^ Since Lf is a natural protein in milk, it offers a high biocompatibility profile, hence lower chances of eliciting adverse immune reactions. Highly proliferating cancer cells have increased iron demand, leading to overexpression of transferrin receptors on tumour cells, including retinoblastoma cells.^139^ In addition, the positively charged lactoferrin can enter the cells *via* electrostatic interaction with the negatively charged cell surface. By virtue of its affinity to receptors overexpressed by cancer cells, Lf could be explored as a targeting moiety for drug-loaded nanocarriers. Moreover, Lf possesses a plethora of pharmacological actions, including antitumor, anti-inflammatory, immunomodulatory, wound healing and antimicrobial activities.^140–142^

Lactoferrin conjugated nanoparticles were developed for enhanced targeting of carboplatin to retinoblastoma cells.^143^ The MTT, DNA laddering assay and caspase-III assays showed that carboplatin-loaded nanoparticles exhibited significantly greater intracellular uptake. Results of pH-dependent-drug release and receptor-blocking assay confirmed the involvement of receptor-mediated endocytosis in the cellular uptake of the nanoparticle. Peptides such as biotinylated peptides have been used as ligands to target retinoblastoma cells. Poly(lactic-*co*-glycolic acid) nanoparticles modified with biotinylated peptides and evaluated for delivering melphalan to the retina *via* the intravitreal route demonstrated more efficacy than unmodified carriers.^144^

Another biomarker investigated for targeted drug delivery in cancer therapy is epithelial cell adhesion molecule (EpCAM). EpCAM is a 40 000 molecular weight (MW) type I transmembrane glycoprotein with low expression levels in normal epithelial cells but over-expressed (up to 1000-fold) in cancer epithelial cells. It is expressed mainly on the basolateral membrane of normal cells, while the expression pattern in cancer cells is mostly on the apical surface.^145^ This differential expression in amount and location makes EpCAM an attractive cancer ligand for targeted drug delivery.

EpCAM antibody modified nanoparticles have been employed in gene delivery to retinoblastoma cells. Mitra *et al.* investigated the use of polyethyleneimine (PEI) gold nanoparticles conjugated with EpCAM antibodies to precisely deliver small interfering RNA (siRNA) to EpCAM-expressing retinoblastoma cells, preventing untoward side effects on normal cells.^146^ Intracellular internalization of the nanoparticles and a remarkable reduction in cell viability of Y79 cells were reported. Compared with the unconjugated PEI gold nanoparticles, EpCAM conjugated PEI gold nanoparticles significantly downregulated the EpCAM gene in the retinoblastoma Y79 cells, as shown by western blot analysis and real-time quantitative PCR results. With greater uptake and enhanced gene silencing efficacy, the EpCAM gold nanoparticle holds great potential in retinoblastoma gene therapy.

Apart from the receptor targeted approach, drug targeting can also be achieved by triggering the release of a chemotherapeutic drug from an environment-responsive smart nanocarrier using a local stimulus (such as low pH, hypoxia, hyperthermia, enzymes and magnetic field).^147^ Uncontrolled cell proliferation and angiogenesis during the growth of cancerous cells result in the formation of tumour microenvironments. These consist of many cells, such as cancer stem cells, endothelial cells, fibroblasts and dendritic cells, which interact with tumour cells to sustain cell proliferation and cancer progression.^148^ In addition to potentiating cell proliferation, they aid in evading apoptotic cell death, facilitating angiogenesis, avoiding growth suppressors and triggering metastasis.^149^ The tumour microenvironment is a significant challenge in cancer therapy. However, it offers a platform for targeted drug delivery to cancer cells. Nanocarriers that respond to acidic pH, excessive ROS and a hypoxic environment and those that can induce hyperthermia have been investigated in this regard.^88,150^

The pH of the tumour microenvironment is lower than that of normal cells and blood, and this can be exploited in targeting chemotherapeutic agents to cancer cells. Anticancer agents are conjugated on nanoparticle surfaces using labile acid bonds, which are stable at neutral physiological pH but easily hydrolyzed in an acidic tumour environment to release the attached anticancer agent. To target deferasirox to cancerous cells, a pH-responsive polyamidoamine dendrimer grown into mesoporous silica nanoparticles and surface modified with glucuronic acid was employed.^88^ The nanocarrier retained the drug at neutral pH and exhibited maximum drug release at the lysosomal pH of 4.5. Thus, the pH-responsive nanoparticles could avoid the premature release of the chemotherapeutic drug in normal cells. On entering tumour cells, deferasirox could be released into the lysosome of tumour cells resulting in enhanced controlled drug delivery. The positive charge surface and the glucuronic acid conjugation of mesoporous silica nanoparticles also enhanced the cell uptake and cytotoxic effect on retinoblastoma cells. This multifunctional system offers targeted and controlled release advantages with limited systemic side effects.

Functionalized nanoparticles can be used to target chemotherapeutic drugs to cancer stem cells (CSCs), a number of cells in a tumour microenvironment with self-renewal and differentiation properties like stem cells. It has been reported that CSCs in retinoblastoma cells cause drug resistance and tumour recurrence after chemotherapy.^151^ They induce cell cycle arrest resulting in a quiescent state. Their ability to go into a quiescent state confers drug resistance on CSCs, as most cytotoxic drugs target proliferating cells.^152^

Lactoferrin nanoparticles loaded with chemotherapeutic drugs, etoposide and carboplatin were used to target Rb Y79 CSCs.^153^ Increased drug uptake and sustained intracellular drug retention were observed in CSCs and non-CSCs. Compared with the standard drug, the cytotoxicity was enhanced in Rb Y79 CSCs compared to non-CSCs. This enhancement in drug uptake, retention and cytotoxicity of the lactoferrin nanoparticles on retinoblastoma Y79 CSCs and non-CSCs is an indication that the functionalized nanoparticle has a promising application in the reversal of chemoresistance and avoidance of cancer recurrence after treatment.

The co-fabrication of a therapeutic agent and an imaging agent (theranostic approach) is an emerging field in cancer therapy inspired by active targeting provided by ligand-functionalized nanoparticles. A folic acid-modified phase-changeable nanoparticle loaded with liquid perfluorobutane and indocyanine green was investigated as a potential theranostic molecular probe.^154^ The surface modification of the nanoparticles with folate was employed to target the probe exclusively to the cancerous cells, thereby reducing adverse effects on the surrounding healthy tissue. The folate receptor targeted theranostic probe demonstrated an enhanced therapeutic effect and improved gene transfection compared with the unmodified nanoparticle *in vitro* and *in vivo*. The photoacoustic and ultrasound contrast shown by the liquid–gas changeable nanoparticle upon laser irradiation was excellent for ultrasound–photoacoustic dual-mode imaging.

Das *et al.* explored the theranostic potential of polymeric nanoparticles loaded with nutlin-3a and modified with EpCAM aptamer and quantum dots (an imaging agent).^155^ Aptamers are single-stranded DNA or RNA oligonucleotides characterized by small size, low immunogenicity and high affinity for EpCAM. The therapeutic and imaging potential of the nanocarrier system was studied in many EpCAM and non-EpCAM-expressing solid cancer cell lines. The study demonstrated the superior therapeutic power of EpCAM-targeted nanoparticles over other formulations in EpCAM-expressing cells. Furthermore, excellent imaging was obtained with the targeted nanocarrier in a 2D monolayer culture and 3D spheroid model. The findings suggest that the nanocarriers may act as a multimodal vehicle capable of enhancing cancer therapy and imaging in diverse cancer types, including retinoblastoma Y79 cells.

Recent studies on the use of ligand-modified nanoparticles in the diagnosis and treatment of retinoblastoma are shown in Table 3.

**Table 3:** Ligand-targeted nanoparticles recently investigated for the diagnosis and treatment of retinoblastoma^a^

Cargo	Mechanism of action	Nanoparticle	Ligand	Biomarker	Zeta potential (mV)	Ref.
Small interfering RNA	Gene silencing	Gold nanoparticles	EpCAM antibody	EpCAM	1.6–2.1	146
Nutlin-3a	MDM2 antagonist/modulator of MDR proteins	Polymeric nanoparticles	Folic acid	Folate receptor	−15.9	133
Curcumin						
Etoposide	Topoisomerase 1I inhibitor	Polymeric nanoparticles	Galactose	Sugar receptors (lectins)	+25	66
Camptothecin/two-photon excitation photodynamic therapy	Topoisomerase 1 inhibitor/formation of reactive oxygen species	Mesoporous silica nanoparticles	Mannose	Mannose or galactose receptors	—	89
Topotecan	Topoisomerase 1 inhibitor	Mesoporous silica nanoparticles	Folic acid	Folate receptor	—	87
Deferasirox	Tridentate iron-chelating drug	Dendritic mesoporous silica nanoparticles	Glucuronic acid	Glucose transporter protein	+5.3	88
Carboplatin	Alkylating agent	Protein nanoparticles	Lactoferrin and apotransferrin	Transferrin and lactoferrin receptor	−23 and −10	143
Carboplatin and etoposide	Alkylating agent/topoisomerase 1I inhibitor	Protein nanoparticles	Lactoferrin	Cancer stem cells	—	153

a EpCAM – epithelial cell adhesion molecule, MDM2 – murine double minute, MDR proteins – multidrug resistance proteins.

## 6. Challenges and future prospects

Some of the challenges encountered by researchers and formulation scientists in the use of nanoparticles for diagnosing and treating retinoblastoma are the ocular barriers, scalability, reproducibility and toxicity of the formulations. The anatomical structure of the eye hinders the entry of bioactive compounds. The presence of ocular blood–retinal and blood–aqueous barriers makes it difficult for the drug to permeate the target cells from systemic circulation. Permeation enhancers and mucoadhesive materials such as chitosan and its derivatives have been widely investigated in this respect. The use of cell penetrating peptides, which have the ability to translocate through the cell membrane and improve cellular uptake, is being investigated for the delivery of genes and drugs to retinoblastoma cells. These peptides conjugated with melphalan have been reported to prevent metastases.^156^ The main area of interest is their applications as a non-invasive means of delivering drugs, small molecules and genes to the retina *via* the topical route. The release and accumulation of chemotherapeutic agents in intraocular tissues *via* green light-responsive nanoparticles is a promising area in retinoblastoma therapy. Green light irradiation at 505 nm can activate systemically administered nanocarriers to release an encapsulated anti-cancer agent specifically to the retina without causing significant harm to the ocular tissues.^157^

The translation of nanoparticles into clinical use is hindered by the non-reproducibility of the system, which leads to batch-to-batch variations of the products. A slight change in the experimental parameters, handling, storage time and environmental factors can significantly affect the physicochemical properties of these systems. Using the design of experiment methodology to formulate and optimize nanoparticles can help overcome this issue.

In the development of a drug product, the preclinical assessment of safety and efficacy is paramount. The two models available for retinoblastoma are xenografts and transgenic mouse models. Efforts are geared towards developing relevant animal models that will simulate human disease, especially for rare diseases such as retinoblastoma with a small number of patients for large clinical trials. Models that reflect all the features of retinoblastoma will aid in understanding the biological mechanism of the disease, thereby reducing the differences in the preclinical and clinical data. A better understanding of the tumour microenvironment is imperative for effective cancer therapy as it has been implicated in multidrug resistance.

Future research on the treatment of retinoblastoma, just like all other oncological diseases, will continue to focus on the local administration of active compounds to suppress the exposure of the patients to possible adverse effects that occur with systemic administration of chemotherapeutic agents. The benefits of non-invasive, posterior segment-specific and sustained-release novel drug delivery systems cannot be overemphasized. Various nanocarriers and nanotechnology-based methods, as well as different materials, are being tried to achieve local administration.^158^ These systems will particularly shut out the possibility of intraocular and extraocular tumour seeding, thereby significantly reducing the risk of metastasis and greatly improving the prognosis of treatment.

There is an emerging trend in exploring smart nanoparticles that would interact specifically with the tumour cells, increasing drug targeting and overcoming the problems of non-specific distribution. These nanoparticulate systems can respond to low pH, hypoxia, hyperthermia, enzymes and magnetic field, which characterize the tumour cells. In addition, the targeting of multiple tumour biomarkers should be exploited to overcome the problems of cancer cell heterogeneity. Moreover, there is a paucity of data on the use of monoclonal antibodies for targeting retinoblastoma cells. This area should be explored as many monoclonal antibodies have been approved for clinical use and are considered standard treatments for some cancers. Utilizing multifunctional nanoparticles with therapeutic and imaging capabilities is another emerging area in diagnosing and treating retinoblastoma. In addition, combinational therapy of chemotherapy and photodynamic therapy has prospects in retinoblastoma therapy. Furthermore, nanomedicines designed to contain more than one drug will be beneficial to the prognosis of retinoblastoma as it may encourage patients' compliance since the current treatment of this disease requires a combination of drugs.^159^

Optogenetic therapy is an emerging approach for treating retinal diseases. It involves the use of light to activate neurons or cells to express a light-sensitive protein with the potential to restore vision. Ding *et al.* employed a nanoparticle-based optogenetic system as an effective and safe option in treating retinoblastoma.^160^ There are ongoing clinical trials on these systems for reversing visual loss, especially in advanced-stage retinal diseases.^161^ A more recent path being explored in the treatment of retinoblastoma is the use of oncolytic viral vectors. These genetically modified viruses can selectively replicate in tumour cells *via* lysis or induction of systemic antitumor immunity.^65^ In non-cancerous cells, the retinoblastoma tumour suppressor gene (RB1) forms a complex with free E2F transcription factors to inhibit cell proliferation. However, the phosphorylation of RB1 by cyclin-dependent kinases prevents the complexation of RB1 with E2F. Oncolytic viral vectors utilize the dysfunctional RB1 pathway with increased expression of E2F to target tumour cells.^65,162^

Electrospun nanofibers are another frontier research area explored for the treatment of cancers, including retinoblastoma. The major advantages include their unique morphological features that allow for favourable drug loading and the possibility of fabrication into various geometries with different sizes.^158^ There are prospects of achieving controlled and more prolonged drug release kinetics for hydrophilic drugs.

The development of new anticancer drugs that can molecularly target tumour cells is another attractive area being explored in retinoblastoma therapy. The use of nano-formulations of medicinal plant extracts or phytochemicals with less toxic effects on healthy cells could be yet another means of improving the therapeutic outcome and toxicity profile of this system.

Despite the challenges in using nanoparticles in cancer therapy, they have shown excellent prospects for delivery of anticancer drugs, genes and imaging agents for diagnosing and treating retinoblastoma.

## 7. Conclusions

Targeting chemotherapeutic drugs to retinoblastoma cells using nanoparticle drug delivery systems holds great potential. Generally, nanoparticles investigated for targeting retinoblastoma cells demonstrated improved uptake and intracellular internalization, sustained retention, excellent cytotoxicity, enhanced apoptosis and an improved antitumor effect compared to conventional treatment options. This review clearly shows the excellent antitumor activity of nanoparticle-based delivery systems in retinoblastoma. It also confirms the great prospects of nanoparticles that should be harnessed in the diagnosis and treatment of retinoblastoma. The emerging trend of using smart nanoparticles in targeting tumour cells could help in overcoming some of the challenges in the delivery of antitumour and imaging agents. Thus, the development of nanoparticle-based targeted drug delivery systems could improve the diagnosis, increase the safety and efficacy of chemotherapy, improve the quality of life and increase the survival rate for retinoblastoma patients.

## Author contributions

Adaeze Linda Onugwu: conceptualization, investigation, writing – original draft, writing – review and editing; Onyinyechi Lydia Ugorji: writing – original draft; Chinasa A. Ufondu: writing – original draft; Stella Amarachi Ihim: writing – original draft; Adaeze Chidiebere Echezona: writing – original draft; Chinekwu Sherridan Nwagwu: writing – original draft; Sabastine Obinna Onugwu: writing – original draft; Samuel WisdomofGod Uzondu: writing – original draft; Chinazom Precious Agbo: writing – original draft; John Dike Ogbonna: writing – review and editing; Anthony Amaechi Attama: conceptualization, writing – review and editing, supervision.

## Conflicts of interest

There are no conflicts to declare.

## Supplementary Material
